# NEIGHBORHOOD SOCIOECONOMIC DISADVANTAGE AND RECOVERY AFTER HIP FRACTURE: EVIDENCE FROM REAL-WORLD MEDICARE DATA

**DOI:** 10.1093/geroni/igae098.2085

**Published:** 2024-12-31

**Authors:** Jason Falvey, Lindsey Mathis, Na Sun, Amit Kumar

**Affiliations:** University of Maryland School of Medicine, Baltimore, Maryland, United States; University of Maryland Baltimore, Baltimore, Maryland, United States; University of Maryland, Baltimore, Maryland, Maryland, United States; University of Utah, Salt Lake City, Utah, United States

## Abstract

Neighborhood environments—or the exposome—are a powerful influence on function recovery among older adults. This study examines the influence of neighborhood socioeconomic disadvantage on the recovery trajectory of older adults following a fall-related hip fracture. Utilizing Medicare fee-for-service claims data, we identified 52,012 older adults with hip fractures via ICD-9 and ICD-10 codes, and identified fall-related mechanisms using validated E codes in claims. These individuals were linked to the Area Deprivation Index (ADI) using 9-digit ZIP codes, categorizing them into the top 10%, bottom 10%, and middle 80%. Days at home, a validated claims-based measure, was calculated for each participant over 6 months. We employed weighted generalized estimating equations with a Poisson distribution to account for the time-varying probability of death in each post-fracture month, and adjusting for age, sex, medical complexity, time (quadratic), and pre-fracture days at home to estimate rate ratios and predicted mean counts of days at home by ADI group. The cohort’s mean age was 82.1 years (SD = 8.1), with 74% female sex. In adjusted models, individuals in the lowest socioeconomic neighborhoods spent more than 7% fewer days at home (RR=0.93, 95% CI 0.91, 0.94) compared to their counterparts in the wealthiest 10%, translating to nearly 8 more days alive and in facilities—exceeding clinically important differences reported in prior work. The study highlights the need for tailored rehabilitation strategies and community support systems that address the unique barriers encountered by this vulnerable population, promoting equitable recovery outcomes across different socioeconomic contexts.

